# N6-Methyladenosine Role in Acute Myeloid Leukaemia

**DOI:** 10.3390/ijms19082345

**Published:** 2018-08-09

**Authors:** Zaira Ianniello, Alessandro Fatica

**Affiliations:** Department of Biology and Biotechnology “Charles Darwin”, Sapienza University of Rome, 00185 Rome, Italy; zairaianniello1994@gmail.com

**Keywords:** m^6^A, RNA, AML, leukaemia, epitranscriptomics

## Abstract

We are currently assisting in the explosion of epitranscriptomics, which studies the functional role of chemical modifications into RNA molecules. Among more than 100 RNA modifications, the N6-methyladenosine (m^6^A), in particular, has attracted the interest of researchers all around the world. m^6^A is the most abundant internal chemical modification in mRNA, and it can control any aspect of mRNA post-transcriptional regulation. m^6^A is installed by “writers”, removed by “erasers”, and recognized by “readers”; thus, it can be compared to the reversible and dynamic epigenetic modifications in histones and DNA. Given its fundamental role in determining the way mRNAs are expressed, it comes as no surprise that alterations to m^6^A modifications have a deep impact in cell differentiation, normal development and human diseases. Here, we review the proteins involved in m^6^A modification in mammals, m^6^A role in gene expression and its contribution to cancer development. In particular, we will focus on acute myeloid leukaemia (AML), which provides an initial indication of how alteration in m^6^A modification can disrupt normal cellular differentiation and lead to cancer.

## 1. Introduction

Leukaemogenesis is caused by gene mutations and chromosomal aberrations resulting in changes of gene expression and, eventually, alteration of cell growth/differentiation programs [1]. Over the past decades, epigenetic modifications (e.g., DNA methylation and histone modifications) have been shown to play a significant role in this process and are now recognized as targets of therapy for different types of leukaemia and other haematological malignancies [2]. More recently, researchers have identified a new layer of gene expression regulation at the RNA levels that consists of reversible chemical modification of messenger RNAs (mRNAs), which led to the birth of the emerging field of “epitranscriptomics” [3,4]. Among more than 100 chemical modifications that can occur within various type of RNA molecules, N6-methyladenosine (m^6^A) is the most abundant internal chemical modification of mRNA and is the one with the greatest impact on its dynamic regulation. The m^6^A modification is installed by “writers” and removed by “erasers”; in addition, it can recruit specific “reader” proteins. m^6^A modification and the associated regulatory proteins play a critical role in gene expression by affecting different steps of the mRNA life, including splicing, nuclear export, stability and translation [4,5]. The reversible and dynamic nature of m^6^A modification and its ability to fine-tune and coordinate gene expression programs has attracted the interest of many research groups in order to define its contribution to cell differentiation, normal development and human diseases [4]. In particular, it has been shown that deregulation of m^6^A modification alters embryonic stem cell maintenance and differentiation [4]. As acquisition of stem cell properties and defects in cell differentiation are common features of many cancers, this indicates that alterations of m^6^A levels might have an important role in cancer development.

In this review, we describe the mammalian proteins involved in m^6^A modification and the effect of the latter on mRNA expression. In particular, we will focus on the role of m^6^A in acute myeloid leukaemia (AML), which provides an initial indication of how alteration in m^6^A levels can disrupt normal cellular differentiation and contribute to carcinogenesis. 

## 2. m^6^A Writers, Erasers and Readers

**Figure 1:**
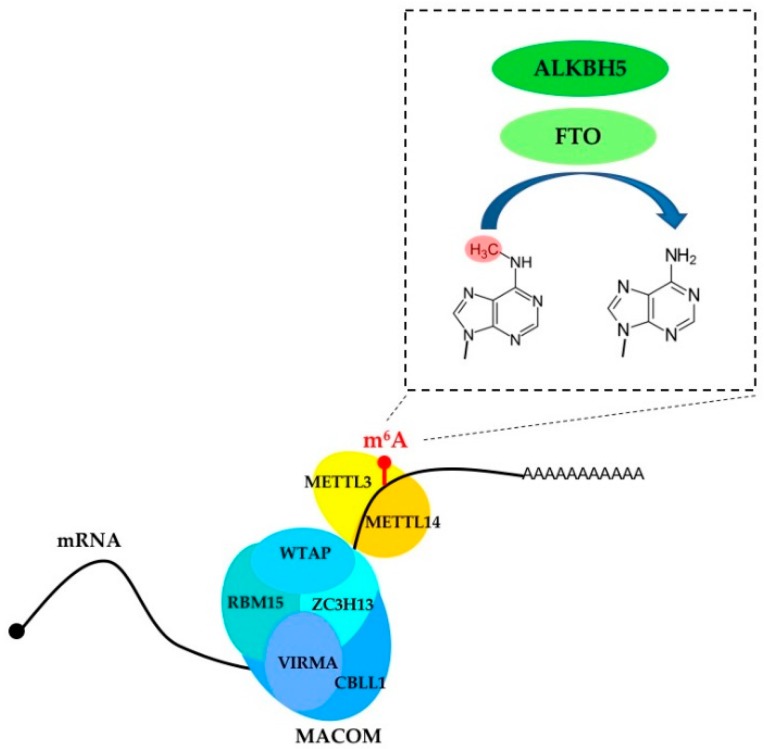
m^6^A modification is installed by the core METTL3/METTL14 (methyltransferase-like protein 3/methyltransferase-like protein 14) catalytic complex and erased by the two demethylases FTO (fat mass and obesity-associated) and ALKBH5 (alkB homologue 5). The MACOM complex, composed of WTAP (Wilms tumor 1-associated protein), RBM15 (RNA-binding motif 15), VIRMA (Vir-like m^6^A methyltransferase-associated), ZC3H13 (zinc finger CCCH-type containing 13) and CBLL1 (Cbl proto-oncogene like 1), guides the core complex on specific mRNAs and contributes to select specific sites within single mRNA.

In mammalian cells, about 0.4% of adenosines inside mRNAs are m^6^A-modified (1–5 m^6^A sites per transcript) [4,5]. The main complex responsible for m^6^A modifications of mRNAs is composed of the methyltransferase-like protein 3 (METTL3) and the methyltransferase-like protein 14 (METTL14). This core heterodimeric complex specifically methylates the adenosine within the DRACH motif (D = A/G/U, R = A/G; H = A/C/U) (Figure 1). METTL3 is the catalytic component of the complex while METTL14 is required for structural stabilization and RNA binding [6,7,8]. In vivo, the activity of METTL3/METTL14 is regulated by an additional complex (referred to as MACOM, m^6^A-METTL-associated complex) composed of Wilms tumor 1-associated protein (WTAP), Vir-like m^6^A methyltransferase-associated (VIRMA, also known as KIAA1429), Cbl proto-oncogene-like 1 (CBLL1, also known as Hakai), RNA-binding motif 15 (RBM15), and zinc finger CCCH-type containing 13 (ZC3H13) proteins [5] (Table 1). Notably, the percentage of m^6^A sites is lower than the occurrence of the consensus motif, indicating that the core methylation complex is specifically recruited by the MACOM complex on specific sites within mRNAs. In particular, modified adenosines are specifically enriched in regions adjacent to the stop codon, 3′-UTR (Untranslated region) and within long internal exons [9,10]. It is very likely that additional cell-specific regulators of m^6^A modification still need to be identified. 

m^6^A modification is essential for embryonic development. Deletion of *METTL3* in mice is embryonic lethal. Moreover, the importance of m^6^A is further shown by the fact that the complete ablation of *METTL3* and *METTL14* in mESCs (mouse embryonic stem cells) impairs the transition of naïve mESCs into the primed state and blocks the subsequent differentiation [11,12]. Similarly, deletion in mice of the regulatory MACOM complex components, *WTAP* and *RBM15*, produced embryonic lethality [13,14,15].

More recently, the U6 snRNA m^6^A methyltransferase-like protein 16 (METTL16) has been shown to target intronic regions of pre-mRNAs and lncRNAs (Long non-coding RNA) [16,17,18]. METTL16 binding sites do not overlap with that one of the METTL3/METTL14 methylation complex, indicating independent functions in m^6^A modification. However, METTL16 plays an important role in regulating the cellular homeostasis of the methyl donor SAM (*S*-adenosylmethionine) [16,17,18], thereby indirectly contributing to global cellular methylation. 

m^6^A marks within transcripts can be removed by the alkB homologue 5 (ALKBH5) and fat mass and obesity-associated (FTO) proteins (Figure 1). These two proteins mainly localized to the nuclear compartment where the removal of m6A modification occurs. FTO has an additional role in demethylating the N6-2′-*O*-dimethyladenosine (m^6^Am) modification close to the mRNA CAP. This modification is installed by a still-unknown modifying enzyme and has an independent role from m^6^A in mRNA stability [19]. In mouse, these two enzymes have different tissue distributions, with FTO being enriched in the brain and ALKBH5 in the testes, suggesting that they can have diverse biological functions and can potentially affect different subsets of target mRNAs. Consistent with their different in vivo expression, *ALKBH5* KO (knock-out) mice show impaired male fertility [20], while *FTO* KO mice exhibit increased postnatal death and reduced body mass [21].

The biological function of m^6^A is largely mediated by m^6^A reader proteins (Table 1). The YT521-B homology (YTH) domain family of proteins (YTHDF1, YTHDF2, YTHDF3, YTHDC1 and YTHDC2), which contain an aromatic cage for specifically accommodating the m^6^A, were among the first to be identified [22]. In addition, m^6^A modification can induce structural alterations in transcripts, which either favor or abolish the interaction of specific RNA binding proteins [23].

## 3. m^6^A Effects on Gene Expression

**Figure 2:**
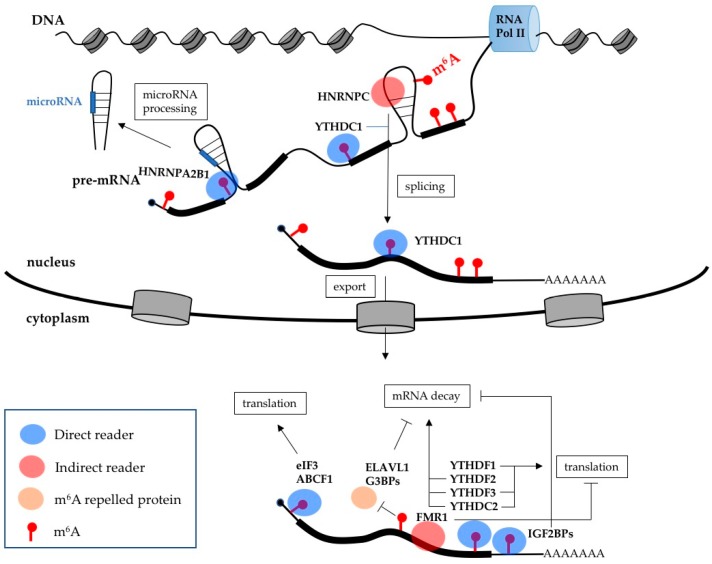
Functional roles of m^6^A modification on mRNA expression. Most of m^6^A’s effects on mRNA metabolism are mediated by reader proteins whose binding can be directly or indirectly affected by m^6^A. In the nucleus, the direct readers are YTHDC1 and HNRNPA2, which stimulate splicing and microRNA processing, respectively. YTHDC1 also stimulates mRNA export. The splicing regulator HNRNPC (heterogeneous nuclear ribonucleoprotein) is an indirect reader whose binding is favored by structural rearrangement induced by m^6^A modification. In the cytoplasm, mRNA translation is stimulated by the direct reader eIF3, ABCF1 (ATP binding cassette subfamily F member 1), YTHDF1, YTHDF3 and YTHDC2, while it is inhibited by the indirect reader FMR1. mRNA decay is increased by the direct readers YTHDF2, YTHDF3 and YTHDC2, while it is inhibited by the direct readers IGF2BPs (insulin-like growth factor 2 mRNA-binding protein) and the m^6^A-repelled proteins ELAVL1 (ELAV like RNA binding protein 1) and G3BPs (G3BP stress granule assembly factor).

m^6^A mediates its biological effects by influencing mRNA synthesis and function (Figure 2). Different biological functions of m^6^A modification are carried out by readers. During transcription, m^6^A is deposited on nascent RNA near splice junctions and within intronic regions [24,25]. The co-transcriptional nature of the m^6^A modification was also confirmed by CLIP (cross-linked immunoprecipitation) experiments in which METTL3 and METTL4 were found associated with intronic regions [26]. Due to its dynamic feature, the distribution of m^6^A peaks is cell type specific and, eventually, also depends on developmental stages and changes in the environment. The core m^6^A methylation complex and the regulatory MACOM complex localize predominantly in nuclear speckles, where the mRNA splicing reaction occurs. Indeed, m^6^A levels modulate alternative splicing by direct and indirect mechanisms. The nuclear reader YTHDC1 directly binds to m^6^A modified pre-mRNAs and regulates splicing by recruiting the SR protein SRSF3 (Serine and arginine-rich splicing factor 3). By contrast, the splicing regulator SRSF10 is repelled by m^6^A modified regions [27]. The hnRNP protein HNRNPA2B1 also mediates alternative splicing by directly binding m^6^A modified pre-mRNA [28]. HNRNPA2B1 binding also stimulates the processing of microRNAs from host pre-mRNA introns [28,29]. In addition, m^6^A modified regions can undergo conformational changes, referred to as an “m^6^A switch”, favoring the interaction with RNA binding proteins, as in the case of the splicing regulator HNRNPC [30]. 

Another crucial step of gene expression that is regulated by m^6^A is mRNA nuclear export. Silencing of METTL3 delays mRNA export, while downregulation of ALKBH5 has the opposite effect and accelerates export [20,31]. The nuclear reader YTHDC1 plays a role in this process recruiting the mRNA export receptor NXF1 (nuclear RNA export factor 1) through SRSF3 [32]. Once in the cytoplasm, m^6^A-modified mRNA is mainly regulated by the YTH reader proteins. In particular, YTHDF1 promotes translation, while YTHDF2 stimulates mRNA decay by recruiting the translation initiation factor eIF3 and the CCR4-NOT deadenylase complex, respectively [33,34]. Interestingly, YTHDF1 and YTHDF2 share a large set of common target mRNAs. These apparently contradictory effects of the two readers may be required for the expression of transcripts that require rapid and transient control, such as in the case of gene expression programs that are activated upon responses to stress and during cellular differentiation. The YTHDF3 reader can cooperate with both YTHDF1 and YTFDF2, thereby promoting both translation and mRNA decay [35,36]. It has been suggested that YTHDF3 contributes to the RNA binding specificity of YTHDF1 and YTHDF2, which eventually bind m^6^A methylated mRNAs as heterodimers [35,36]. Strikingly, YTHDF2 KO mice are embryonically lethal [37]. While deletion of YTHDF1 does not affect embryo development and it is compatible with life [38].

YTHDC2 has a dual role in controlling the expression of m^6^A modified mRNAs, it both enhances their translation and accelerates their decay [39,40,41]. In contrast to the other YTH proteins, YTHDC2 contains other functional regions in addition to the YTH domain, including an ATP-dependent RNA helicases domain interspersed with two Ankyrin repeats, a known protein–protein interaction module that is responsible for the recruiting of the Xrn1 exoribonuclease [39,40,41]. In mouse, the protein is highly expressed in germ cells and is essential for male and female fertility. It has been suggested that the binding of YTHDC2 can accelerate protein synthesis, as well as rapid mRNA decay, to timely regulate gene expression during cell differentiation and developmental programs [39,40,41].

Additional direct m^6^A cytoplasmic readers, which lack the YTH domain, are the translation initiation factor eIF3 and the ABCF1 (ATP binding cassette subfamily F member 1) proteins, which stimulate CAP-independent translation of mRNA m^6^A-modified in the 5′-UTR [42,43,44], and the IGF2BPs (insulin-like growth factor 2 mRNA-binding protein) (IGFBP1, 2 and 3) oncogenic proteins [45], which increase the stability of modified mRNAs. Thus, the latter have an opposite role to the YTH reader proteins. Moreover, binding sites for IGF2BPs present a different pattern compared to the YTH proteins because are enriched in the 3′-UTR. Finally, the negative regulators of translation FMR1, and its paralogues FXR1 and FXR2, are indirect readers of m^6^A modified transcripts. Conversely, the G3BP1, G3BP2 (G3BP stress granule assembly factor) and ELAVL1 (ELAV-like RNA binding protein 1) (also known as HuR) RNA binding proteins, which stabilize mRNA upon binding, are indirectly repelled by the m^6^A modification [23,46]. Altogether, these data show the existence of a complex network of interactions between m^6^A modification and RNA-binding proteins that can regulate mRNA expression at multiple levels.

## 4. m^6^A Roles in AML (Acute Myeloid Leukaemia) and Normal Haematopoiesis

Defects in cell differentiation and uncontrolled proliferation are a hallmark of several cancers. AML represents a remarkable example of malignancy with these features [1]. It is characterized by an accumulation of immature leukemic cells (also referred to as blasts) in the bone marrow and blood. This accumulation arises from a failure of myeloid progenitors to mature and respond to normal regulators of proliferation. Chromosome aberrations, such as translocations, inversions and deletions, are detectable in about half of AML patients and are utilized for the classification and as prognostic factors of the disease. Moreover, a number of gene mutations as well as deregulated expression of genes have been identified and has provided insights into the mechanisms of leukaemogenesis [1]. Noteworthy, cells derived from different AML subtypes can be induced to differentiate by specific agents into cells that resemble normal counterparts. For this reason, AML cells have been extensively utilized for studying the molecular mechanisms that regulate the correct balance between proliferation and differentiation.

### 4.1. WTAP (Wilms Tumor 1-Associated Protein) in AML

First clue of the involvement of the m^6^A modification in cancer was the identification of WTAP protein as specific interactor of the Wilms’ tumor gene (*WT1*) [47], even if at that time the complex responsible for the m^6^A modification and its role in mRNA metabolism were still unknown. *WT1* was initially discovered as a tumor suppressor gene, but in leukaemia, where it is generally overexpressed and associated with poor prognosis, it acts as an oncogene [48]. Later on, WTAP protein was found up-regulated in AML and its downregulation in AML cell lines decreased proliferation, induced apoptosis and delays leukaemia progression in recipient mice [49]. At the same time, WTAP was identified as a regulatory factor for the m^6^A methylation complex [50], at which point m^6^A modification becomes the focus of AML studies. 

### 4.2. Core METTL3/METTL14 (Methyltransferase-Like Protein 3/Methyltransferase-Like Protein 14) Complex in AML

Notably, AML is one of the cancers with the highest levels of both METTL3 and METTL14 expression (data from the Cancer Genome Atlas, TCGA) and, more importantly, METTL3 and METTL14 were found overexpressed in AML cells compared to normal haematopoietic progenitors [51,52,53,54]. Consistent with *METTL3* and *METTL14* playing an oncogenic role in AML, overexpression of both genes in AML cell lines and primary blasts increased proliferation, while their downregulation impaired proliferation and resulted in a strong induction of apoptosis [51,52,53]. The oncogenic function of m^6^A was also demonstrated in primary cells derived from an AML mouse model carrying the oncogenic *MLL-AF9* fusion gene and the *FLT3* internal tandem duplication (FLT3-ITD), two chromosomal translocations that characterized aggressive AML subtypes and that drive AML in mouse. In this case, a genome-wide CRISPR/Cas9 screening identified *METTL3, METTL14* and *METTL16* as critical genes for AML survival [52]. More importantly, the METTL3/METTL14 methylation complex was found to promote the development of AML and maintain leukaemia-initiating cells in transplantation mouse models [51,52,53]. These effects of METTL3 and METTL14 in human and mouse AML cells are abolished by catalytic inactive METTL3 or METTL14 mutant impaired in target recognition of the methyltransferase complex, therein, they depend on the deposition of m^6^A modification. In AML cells, METTL3 and METTL14 bound predominantly to transcription start sites, even if METTL3 binding did not always correlate with METTL14, and early m^6^A co-transcriptional deposition promoted translation of mRNAs relevant for AML proliferation, such as c-MYC, BCL2, PTEN, SP1 and MYB [51,52,53]. The transcription factor CEBPZ (CCAAT enhancer binding protein), which as an important role in haematopoietic differentiation, was shown to recruit METTL3 on gene promoters [52]. CEBPZ was identified as a novel recurrently mutated gene in AML [55,56], suggesting that in this leukaemia might be lost the co-transcriptional recruitment of METTL3. 

Interestingly, even if aberrant alternative splicing plays a relevant role in AML [57] and m^6^A modification has an established role in regulating alternative splicing, mRNA translation has been identified as the main regulatory step deregulated by m^6^A in AML [51,52,53]. Therein, the YTHDF1 reader should mediate most of the observed phenotype in AML. However, in view of the pleiotropic nature of the m^6^A modification, it is very likely that additional functions for the other m^6^A readers will be soon characterized in AML.

In AML, METTL3 has been also shown to be mis localized to the cytoplasm and to associate with translating ribosomes [54]. Similar results were also found in lung cancer [58]. It was also demonstrated that cytoplasmic METTL3 can promote translation of specific mRNAs independently from its catalytic activity [54,58]. Moreover, higher levels of cytoplasmic METTL3 results in concomitant increase of WTAP protein expression [54]. As WTAP mRNA expression is not elevated in AML [54], this latter mechanism might be relevant to increase WTAP protein levels concomitantly to the METTL3/METTL14 core complex and sustain its oncogenic role in AML.

### 4.3. RBM15 (RNA-binding motif 15) in AML

Another link between m^6^A and AML comes from RMB15, a component of the MACOM complex. Some forms of Acute Megakaryoblastic Leukaemia (AMKL), a subtype of paediatric AML characterized by abnormal megakaryoblasts, carry a chromosomal translocation between RBM15 and MKL1 [59], a transcription coactivator that regulates the expression of genes involved in cell growth. Notably, RBM15 directly binds to and controls the alternative splicing of transcripts encoding for key haematopoietic differentiation genes such as *GATA1*, *RUNX1*, *c-MPL* and *TAL1* [60]. Therein, it is possible to speculate that the RBM15 fusion protein might also affect MACOM function and produce a deregulation of the m^6^A modification within transcriptome that will eventually result in aberrant splicing regulation. Conditional-knockout mice of RBM15 in the haematopoietic compartment have been generated. Deletion of RBM15 caused a block in B cell differentiation and myeloid and megakaryocytic expansion [15], indicating that mis regulation of m^6^A deposition may affect haematopoietic differentiation in vivo. However, the relationship between RBM15 depletion and m^6^A modification in mouse models has not yet been assessed.

### 4.4. FTO (Fat Mass and Obesity-Associated) in AML 

m^6^A levels depend also on the activity of erasers and readers. Elevated expression of FTO has been reported in AML subtypes carrying MLL-AF9, PML-RARA and FTL3-ITD translocation, respectively [61]. Downregulation of FTO in cell models carrying these fusion products decreased their proliferation capacity. Moreover, it was shown that R-2HG (*R*-2-hydroxyglutarate), an anti-leukemic compound that inhibits a series of Fe(II)/a-KG-dependent dioxygenases [62], also targets the FTO demethylase [63]. In particular, FTO inhibition by R-2HG resulted in decreased expression of the MYC oncogene and the transcription factor CEBPA. These results are in sharp contrast with the pro-leukemic roles of METTL3 and METTL14 demonstrated by independent groups, in many cases by using cells with the same mutations in which the oncogenic role of FTO was demonstrated [51,52,53]. Moreover, it was shown that the depletion of FTO by CRISPR/cas9 has no effect on AML growth [51] and the Project DRIVE, a large-scale knockdown screening in cancer cell lines, detected no general FTO-dependency of leukaemia cells [64]. These discrepancies may be due to the conditions used in those studies, rather than a specific oncogenic effect of FTO in AML. It should be also pointed out that there is currently a great debate on the effective contribution of FTO as specific m^6^A demethylases [65].

### 4.5. Core METTL3/METTL14 Complex in Normal Haematopoiesis

METTL3 and METTL14 are also highly expressed in mouse and human HSCs (haematopoietic stem cells), and their expressions decrease during myeloid differentiation [51,52,53]. As observed in AML cell lines, METTL3 and METTL14 silencing in human and mouse HSCs reduced proliferation capacity and stimulated myeloid differentiation [51,52,53]. Moreover, overexpression of a catalytic active METTL3 or METLL14 in HSCs promoted proliferation and inhibited myeloid differentiation [51,52,53], indicating that increased m^6^A levels might alter the normal differentiation pathway in HSC, resulting in accumulation of progenitor cells. 

Downregulation of METTL3 in zebrafish embryos and in the mouse aorta-gonad-mesonephros (AGM), the region of the primary origins of the definitive HSC in vertebrates, strongly affect HSC production by repressing Notch signaling [66]. It was also shown that the embryonic METTL3 function in the haematopoietic system is mediated by the YTHDF2 dependent decay of Notch encoding mRNA [66]. Similar results were obtained upon conditional METTL3 KO in the mouse AGM region [67], therein, indicating an evolutionally conserved function of METTL3 in HSPCs (haematopoietic stem and progenitor cells) specification in vertebrates. Conditional KO mice of *METTL3* and *METTL14* in the adult haematopoietic system have been also produced [53,68]. Strikingly, deletion of *METTL3* expands the HSCs in adult bone marrow [68], contrary to what occurred in isolate HSCs, indicating a crucial role for METTL3 in regulating the quiescence of HSCs in vivo. Surprisingly, this phenotype was not detected upon conditional deletion of *METTL14* [68], which is required for METTL3-mediated m^6^A modification on target RNAs. However, in transplantation experiments, mouse HSCs deleted for *METTL3* or *METTL14* showed reduced repopulation ability and the deletion of *METTL14* from primary leukaemia blasts significantly delayed leukaemia onset in recipient mice [38,68]. Notably, the downregulation of the m^6^A methylation complex in normal HSCs did not induce massive apoptosis as observed in AML [51,53,66,68], even if the molecular mechanism responsible for this different behavior is still not completely clear. In view of this, it is tempting to speculate that AML cells will show higher sensitivity to future chemical inhibitors of the m^6^A writing complex than normal HSCs providing new therapeutic options for AML treatment.

## 5. Conclusions

Advances in the understanding of AML pathogenesis have been remarkable in these years, but treatment has changed little in the past decades and is mainly based on chemotherapy. Moreover, in AML patients, relapse is frequent and generally accompanied by very poor prognosis. For this reason, many studies are now focusing on the development of new treatments that may flank, replace or follow standard therapy. Epigenetic modifications to histones and DNA have established roles in normal haematopoietic development and leukaemia. More importantly, epigenetic drugs that specifically target these modifications are currently in clinical trials for the treatment of AML. Until recently, little attention has been given to understanding the role of RNA chemical modifications and its contribution to human cancer, even if their presence within different RNA species have been known for decades. The explosion of epitranscriptomics is deeply changing our approaches to cancer biology studies. In the last year, we have assisted in many acclaimed revolutions in the RNA field that have deeply transformed our understanding of how gene expression is regulated; however, none of these has led to major innovations in tumor therapies. Even if the road ahead is still long, it appears to be different this time with epitranscriptomics. Currently, alteration of m^6^A levels has been found to be closely associated with various kinds of cancers and to play important roles in metastasis and drug resistance [69,70]. AML has indicated how alteration of m^6^A modification can disrupt normal cellular differentiation and contribute to cancer development. In analogy with epigenetic modifications, it is predictable that the activity of the m^6^A modifying enzymes might be easily targetable by chemical compounds and may, eventually, provide major innovations in future cancer therapies. In this regard, AML is paving the way.

## Figures and Tables

**Table 1:** Mammalian m^6^A (N6-methyladenosine) regulators.

Protein	Classification	Function
METTL3	Writer	Installs m^6^A in mRNA; promotes translation
METTL14	Writer	Cooperates with METTL3 in m^6^A installation
METTL16	Writer	Installs m^6^A in U6 snRNA and pre-mRNA
FTO	Eraser	Remove m^6^A and m_6_Am from mRNA
ALKBH5	Eraser	Remove m^6^A from mRNA
WTAP	Component of the regulatory MACOM complex	Regulates m^6^A installation
VIRMA	Component of the regulatory MACOM complex	Regulates m^6^A installation
CBLL1	Component of the regulatory MACOM complex	Regulates m^6^A installation
RBM15	Component of the regulatory MACOM complex	Regulates m^6^A installation
ZC3H13	Component of the regulatory MACOM complex	Regulates m^6^A installation
ABCF1	Direct reader	Stimulates translation
eIF3	Direct reader	Stimulates translation
HNRPA2B1	Direct reader	Stimulates microRNA processing
IGF2BPs	Direct readers	Increase mRNA stability
YTHDC1	Direct reader	Stimulates splicing and mRNA export
YTHDC2	Direct reader	Stimulates mRNA decay and translation
YTHDF1	Direct reader	Stimulates translation
YTHDF2	Direct reader	Stimulates mRNA decay
YTHDF3	Direct reader	Stimulates mRNA decay and translation
FMR1	Indirect reader	Inhibits translation
HNRNPC	Indirect reader	Regulates splicing
ELAVL1	m^6^A repelled protein	Increases mRNA stability
G3BPs	m^6^A repelled protein	Increase mRNA stability

## Abbreviations

ABCF1	ATP binding cassette subfamily F member 1
AML	Acute myeloid leukaemia
CEBP	CCAAT enhancer binding protein
CLIP	Cross-linked Immunoprecipitation
ELAVL1	ELAV like RNA binding protein 1
G3BP	G3BP stress granule assembly factor
hnRNP	Heterogeneous nuclear ribonucleoprotein
HSC	Haematopoietic stem cell
HSPC	Haematopoietic stem and progenitor cells
IGF2BP	Insulin-like growth factor 2 mRNA-binding protein
KO	Knock-out
LncRNA	Long non-coding RNA
mESC	Mouse embryonic stem cells
R-2HG	*R*-2-hydroxyglutarate
SAM	*S*-adenosylmethionine
UTR	Untranslated region
